# Time trends of cancer incidence in Setif, Algeria, 1986–2010: an observational study

**DOI:** 10.1186/1471-2407-14-637

**Published:** 2014-08-30

**Authors:** Mokhtar Hamdi Cherif, Diego Serraino, Abbes Mahnane, Slimane Laouamri, Zoubida Zaidi, Hafida Boukharouba, Dahbia Cherka, Manel Rakeb, Lamia Kara, Asma Ayat, Silvia Birri, Saverio Virdone, Paolo De Paoli, Ettore Bidoli

**Affiliations:** Faculty of Medicine, University of Setif, Kragujevac, Algeria; Unit of Epidemiology and Biostatistics, Centro di Riferimento Oncologico, IRCCS, via Franco Gallini 2, 33081 Aviano, PN Italy

**Keywords:** Cancer incidence, Time trends, Setif, Algeria, North-Africa

## Abstract

**Background:**

Incidence rates of various cancers are increasing in Arab countries and are expected to reach those of industrialized ones in few decades. This paper aimed to describe the incidence rates of most common cancers - and/or of those cancer preventable through modifiable behaviors - recorded in the province of Setif, Algeria from 1986 through 2010.

**Methods:**

Cancer diagnoses for the 1986–2010 period were provided by the population-based Cancer Registry of Setif, disentangled by site, morphology, age (quinquennia), sex, and calendar period. The corresponding population was obtained from the Algerian Institute of Statistics. Age-standardized rates (world population) (ASR-WR) were computed by calendar period (five quinquennias from 1986–1990 to 2006–2010), while annual percent changes (APCs) were computed for the period 1996–2010.

**Results:**

During the 2006–2010 period, ASR-WR for all cancer sites were 106.4/100,000 in men and 110.3 in women. The four leading cancers were: lung (18.0%); colon-rectum (9.6%); bladder (9.1%); and prostate (6.5%) in men; breast (36.4%); colon-rectum (8.5%); cervix uteri (6.0%); and thyroid (6.0%) in women. Between 1996–2010, overall cancer incidence increased statistically significantly (p < 0.05) in both men (APC = +2.5%) and women (APC = +3.7%). Statistically significant decreasing trends were observed for nasopharyngeal carcinoma (APC = -3.4%) in men, and for cervical (APC = -4.2%) and gallbladder (APC = -3.2%) cancers in women. Statistically significant increasing trends were observed for most common cancers both in men (lung:+1.8%, colon-rectum:+5.4%, prostate:+4.3%, liver:+8.9%, and bladder:+5.9%) and women (breast:+8.2%, colon-rectum:+4.5%, lung:+10.0%, liver:+5.4%, thyroid:+5.3%, and larynx:+13.8%).

**Conclusions:**

International recommendations against cancer must be strongly promoted in Setif after taking into account epidemiological transition, lifestyle, and environmental changes.

## Background

Cancer rates in northern Arab countries lying on the African side of the Mediterranean Sea are substantially lower than those reported in European Mediterranean countries such as Spain, France or Italy [1]. However, some increases in cancer incidence in northern African countries (e.g., Algeria, and Tunisia) have been recently observed for some cancer sites [2–5]. Although still one third or one half lower than those of industrialized countries, incidence rates are expected to rise and to reach similar levels in few decades. Furthermore, variations in overall rates among those countries were by far smaller than the differences between this group of countries and Europe [6]. A substantial proportion of these increasing cancer rates is potentially avoidable by primary and secondary prevention programs, while improvements in diagnosis and anticancer treatments would increase survival. In Algeria, cancer has become a national priority bringing to a 2014–20 national cancer plan under aegis of the President of the Republic.

The cancer registry of Setif (CR-S), Algeria, has been recording incidence data for the whole Wilaya (province) of Setif, the second in size after the Wilaya of Algiers - the capital city of Algeria. An accurate description of temporal trends by cancer site can contribute to a better understanding of cancer incidence patterns and their potential etiology and/or prevention. We took advantage of the availability of computerized incidence data recorded in CR-S over the 1986–2010 period to describe trends in incidence for the most common cancer sites and/or for the potentially preventable ones.

## Methods

Data regarding all incident cancer cases diagnosed during the period 1986–2010 derived from the population-based cancer registry of Setif, established in 1989 in collaboration with the International Agency for Research on Cancer (IARC). The Director of the Cancer Registry of Setif (Prof. Hamdi Cherif) approved the use of the registry data for the purposes of the study. Nowadays, the CR-S covers a population of about 1.5 million inhabitants (as attested by 2008 national census), which is nearly 5% of the Algerian population. Collection of data is primarily active.

For the aims of this analysis, incidence data were disentangled by age (quinquennia), sex and type of cancer. The International Classification of Diseases for Oncology, second version (ICD-O-3), was used for cancer classification [7]. The ICD-O-3 codes were translated to ICD-10 for data analysis. Registration validity and completeness were evaluated using percentage of microscopic verification (MV) index (the proportion of incident cases with histological and/or cytological verification of cancer diagnosis) and percentage of death certificates only (DCO) (the proportion of incident cases with information based on death certificates only). For all cancers the MV was 96.2% (ranging from 93.4% in lung cancer to 98.8% in breast cancer), while the proportion of DCO was 1.2% (ranging from 0.2% for cervical cancer to 3% in leukemias). To improve validity and comparability of some cancer sites/types with other populations that used previous cancer classifications [8, 9], we summed-up case frequencies of intestinal sites, non-Hodgkin’s lymphomas, and leukemias. In particular, the statistical analysis focused on the following cancers, or groups of cancers (ICD-10): nasopharynx (C11), stomach (C16), colon-rectum (C18–21), liver (C22), gallbladder (C23-24), larynx (C32), lung (C33–34), prostate (C61), breast (C50), cervix uteri (C53), corpus uteri (C54), uterus unspecified (C55), ovary (C56), kidney and other urinary sites (C64-66, C68), bladder (C67), thyroid (C73), Hodgkin’s lymphoma (C81), non-Hodgkin’s lymphomas (C82–85, C96), all leukemia (C91–95), unspecified or ill-defined cancers (C26, 39, 48, 58, 76, 77, 80), and all malignant cancers excluding skin carcinoma (C00-97, but C44). Corpus uteri and uteri unspecified were only examined to quantify any classification shift between uterus sub-sites during the study period.

The structure of the resident population by sex, age, and calendar year was abstracted from the Algerian Institute of Statistics database [http://www.ons.dz].

Incidence data were grouped into five quinquennia of calendar years (1986–1990, 1991–1995, 1996–2000, 2001–2005, and 2006–2010). Age-standardized incidence rates per 100,000 (to the world standard population) [10] (ASR-WR) were calculated in both sexes by the five quinquennia described - or for each calendar year from 1996 to 2010 - according to selected cancers or groups of cancers. Incidence rates and their corresponding standard errors were calculated using SEER*Stat (Surveillance Research Program, National Cancer Institute SEER*Stat software, seer.cancer.gov/seerstat, version 8.1.2).

The computation of annual percent change (APC) [11, 12] of incidence rates was restricted to the 1996–2010 period, to specifically quantify the recent impact of cancer incidence on the Setif population. APCs were calculated separately by age groups (15–44 years, 45–64 years, and 65+ years) and in the total population. APCs were estimated by fitting a linear regression line to the natural logarithm of annual incidence rates using calendar year as a regressor variable. This calculation assumes that the incidence rates changed at a constant rate over the entire calendar-year interval examined, and the validity of this assumption was checked by merely examining plotted curves. APCs were computed using the Joinpoint software and their statistical significance (p < 0.05) was calculated by means of a Student’s t-distribution [12].

## Results and discussion

The overall number of incident cancers (all cancers, excluding skin carcinoma) diagnosed in the Wilaya of Setif during the period 2006–2010 was 6235 (2883 men and 3352 women) corresponding to an ASR-WR (per 100,000) of 106.4 in men and 110.3 in women (Table 1). Nearly 70% of overall cancer incidence was attributable in both sexes, to few leading cancers, namely: lung (18.0%); colon-rectum (9.6%); bladder (9.1%); prostate (6.5%); stomach (5.5%); non-Hodgkin’s lymphomas (5.5%); larynx (5.3%); nasopharynx (5.0%); and leukemias (4.8%) in men. In women, the major sites were breast (36.4%); colon-rectum (8.5%); cervix uteri (6.0%); thyroid (6.0%); gallbladder (4.4%); lung (3.6%); non-Hodgkin’s lymphomas (3.3%) and leukemias (3.2%) (Table 1). By contrast, these cancers explained a lower proportion of overall cancers during the period 1986–90 (64% in men and 57% in women).

**Table 1:** **Number of incident cases and age-adjusted standardized rates (world population) (ASR-WR) of selected cancers by sex according to calendar period (quinquennia), Sétif Cancer Registry**

Cancer or group of cancers	Period										
	1986-1990		1991-1995		1996-2000		2001-2005		2006-2010		
	N	ASR-WR/10 ^5^	N	ASR-WR/10 ^5^	N	ASR-WR/10 ^5^	N	ASR-WR/10 ^5^	N	%	ASR-WR/10 ^5^
**Men**											
Nasopharynx	78	4.5	121	5.9	169	7.3	170	6.4	144	5.0	4.8
Stomach	146	9.8	131	8.1	143	7.0	142	6.6	159	5.5	6.1
Colon-rectum	33	2.1	58	3.4	136	5.9	189	8.1	277	9.6	9.9
Liver	60	4.0	36	2.3	11	0.6	35	1.7	53	1.8	2.1
Gallbladder	30	2.0	32	2.0	30	1.6	60	2.9	52	1.8	2.1
Larynx	45	3.1	55	3.6	98	5.3	137	6.7	153	5.3	5.9
Lung	181	12.7	242	15.8	308	17.1	414	20.2	518	18.0	21.1
Prostate	25	1.7	39	2.6	90	5.1	101	4.8	188	6.5	7.6
Kidney	7	0.3	12	0.7	17	0.7	26	1.2	26	0.9	0.9
Bladder	25	1.6	31	1.9	100	5.7	164	8.1	262	9.1	10.3
Thyroid	5	0.3	5	0.2	23	1.0	31	1.2	43	1.5	1.4
Hodgkin lymphoma	43	1.8	51	2.1	42	1.4	103	3.3	67	2.3	1.9
Non Hodgkin lymphomas	60	3.3	80	3.9	123	5.1	107	4.0	158	5.5	5.2
Leukaemias	119	5.1	92	3.7	52	2.2	63	2.4	139	4.8	4.6
Unspecified and ill-defined cancers	86	5.4	70	3.6	85	4.0	76	3.0	119	4.1	4.5
All cancers but skin	1118	67.9	1230	69.3	1683	82.2	2136	93.3	2883	100	106.4
**Women**											
Nasopharynx	41	2.0	60	2.7	49	1.7	63	2.2	69	2.1	2.1
Stomach	79	4.8	57	3.2	64	2.7	81	3.4	80	2.4	2.8
Colon-rectum	39	2.4	59	3.0	142	6.1	213	8.6	285	8.5	9.9
Liver	80	5.1	34	2.0	18	0.9	26	1.1	44	1.3	1.6
Gallbladder	100	6.7	150	8.8	168	8.4	199	9.0	149	4.4	5.7
Larynx	7	0.4	2	0.1	3	0.1	13	0.5	29	0.9	0.9
Lung	30	2.0	34	2.0	38	1.7	50	2.2	121	3.6	4.5
Breast	192	10.6	253	13.6	440	20.2	571	23.1	1221	36.4	39.5
Cervix uteri	156	9.3	221	12.3	222	11.3	239	10.7	201	6.0	7.1
Corpus uteri	13	0.9	16	1.0	26	1.4	6	0.3	8	0.2	0.3
Uterus. unspecified	12	0.8	1	0.1	5	0.3	14	0.6	39	1.2	1.5
Ovary	26	1.4	45	2.3	61	2.5	30	1.1	75	2.2	2.2
Kidney	14	0.6	8	0.3	28	1.0	17	0.7	27	0.8	1.0
Bladder	3	0.2	2	0.1	4	0.2	50	2.2	34	1.0	1.2
Thyroid	22	0.9	31	1.5	81	3.6	145	5.4	200	6.0	6.2
Hodgkin lymphoma	24	0.9	32	1.1	29	0.9	63	1.6	78	2.3	1.9
Non Hodgkin lymphomas	35	1.7	46	2.1	79	3.0	90	3.3	112	3.3	3.4
Leukaemias	77	3.5	76	3.1	50	1.9	56	2.1	107	3.2	3.1
Unspecified and ill-defined cancers	77	4.2	51	2.5	76	3.4	76	3.0	117	3.5	4.1
All cancers but skin	1143	64.4	1292	67.0	1727	77.9	2190	87.8	3352	100	110.3

Throughout the study period (25 years), overall cancer incidence rates steadily increased in both sexes (Table 1). In men, rates (per 100,000) increased from 67.9 in 1986–1990 to 106.4 in 2006–2010 while in women rates increased from 64.4 in 1986–90 to 110.3 in 2006–2010. Between 1996 and 2010 (last 15 years of observation), APCs (% per year) increased statistically significantly (p < 0.05) by +2.5 (95%CI:+1.6;+3.4) in men, and by +3.7 (95%:+2.6;+4.8) in women (Table 2). These positive APCs were consistent across all age groups (15–44 years, 45–64 years, and 65+ years) with statistically significant (p < 0.05) APCs in the three age groups in women and in the 65+ age group in men.

**Table 2:** **Annual Percent Changes (APC), and corresponding 95% Confidence Intervals (CI), of selected cancers by sex according to age groups, during the period 1996-2010, Sétif Cancer Registry**

Cancer or group of cancers	All Ages		15-44 years		45-64 years		65+ years	
	APC	95% CI	APC	95% CI	APC	95% CI	APC	95% CI
**Men**								
Nasopharynx	-3.4^*^	(-5.1;-1.6)	-4.9^*^	(-8.5;-1.1)	-4.0^*^	(-6.8;-1.1)	-2.4	(-9.8;+5.6)
Stomach	-1.1	(-2.7;+0.6)	-7.0^*^	(-12.0;-1.8)	-2.0	(-5.9;+2.2)	+2.7	(-0.6;+6.1)
Colon-rectum	+5.4^*^	(+3.4;+7.6)	-0.8	(-4.7;+3.3)	+3.7^*^	(+0.5;+7.0)	+11.1^*^	(+6.5;+15.8)
Liver	+8.9^*^	(+0.9;+17.5)	+3.6	(-13.8;+24.6)	+1.6	(-7.8;+12.1)	+9.6	(-0.4;+20.6)
Gallbladder	+1.0	(-5.2;+7.6)	-4.3	(-15.3;+8.0)	-0.2	(-8.2;+8.5)	+3.3	(-4.8;+12.1)
Larynx	+1.0	(-4.8;+7.1)	+4.7	(-5.9;+16.4)	-4.8	(-10.8;+1.6)	+5.1	(-0.9;+11.4)
Lung	+1.8^*^	(+0.0;+3.6)	+4.7	(-3.8;+14.0)	+0.7	(-1.6;+3.1)	+2.8^*^	(+0.3;+5.4)
Prostate	+4.3^*^	(+2.0;+6.8)	+1.0	(-18.4;+25.0)	+0.9	(-3.7;+5.8)	+5.0^*^	(+1.7;+8.5)
Kidney	+4.3	(-1.1;+10.0)	+0.6	(-14.8;+18.7)	+2.0	(-5.5;+10.1)	-0.6	(-10.6;+10.6)
Bladder	+5.9^*^	(+4.1;+7.7)	+7.4	(-1.1;+16.5)	+3.5^*^	(+0.4;+6.6)	+8.0^*^	(+4.7;+11.5)
Thyroid	+3.2	(-3.6;+10.5)	+0.1	(-7.6;+8.5)	-0.2	(-9.1;+9.6)	+2.6	(-7.2;+13.5)
Hodgkin lymphoma	-0.6	(-7.8;+7.2)	+1.2	(-6.6;+9.6)	-1.9	(-11.1;+8.4)	+5.4	(-8.0;+20.8)
Non Hodgkin lymphomas	+0.8	(-3.3;+5.0)	+2.3	(-2.8;+7.7)	-1.8	(-8.4;+5.4)	+4.4	(-2.7;+11.9)
Leukaemias	+6.7	(-0.4;+14.2)	+13.7^*^	(+6.8;+21.0)	+3.0	(-3.0;+9.4)	+6.0	(-4.2;+17.2)
Unspecified and ill-defined cancers	+0.5	(-4.2;+5.5)	-0.6	(-9.1;+8.6)	-1.1	(-6.5;+4.6)	+4.4	(-1.7;+10.9)
All cancers but skin	+2.5^*^	(+1.6;+3.4)	+1.5	(-0.4;+3.4)	+0.9	(-0.4;+2.2)	+5.1^*^	(+3.8;+6.4)
**Women**								
Nasopharynx	+2.5	(-1.6;+6.7)	-0.7	(-6.1;+4.9)	+5.5	(-1.6;+13.1)	+0.0	(-6.7;+7.1)
Stomach	+0.6	(-1.7;+2.9)	-6.6^*^	(-12.4;-0.4)	+0.3	(-4.5;+5.4)	+3.3	(-1.8;+8.6)
Colon-rectum	+4.5^*^	(+1.6;+7.4)	-2.1	(-6.3;+2.3)	+5.3^*^	(+1.4;+9.3)	+7.5^*^	(+3.8;+11.3)
Liver	+5.4^*^	(+0.2;+10.8)	+6.6	(-6.0;+20.8)	+5.3	(-2.5;+13.8)	+3.1	(-4.0;+10.8)
Gallbladder	-3.2^*^	(-5.8;-0.5)	-8.7^*^	(-14.5;-2.5)	-2.8	(-5.8;+0.4)	-2.1	(-6.6;+2.7)
Larynx	+13.8^*^	(+0.6;+28.7)	+22.8	(-1.3;+52.9)	+6.7	(-7.7;+23.3)	+5.4	(-9.9;+23.2)
Lung	+10.0^*^	(+5.9;+14.2)	+6.2	(-0.8;+13.6)	+10.4^*^	(+3.2;+18.1)	+7.8^*^	(+2.8;+13.1)
Breast	+8.2^*^	(+5.7;+10.7)	+9.7^*^	(+5.8;+13.7)	+7.3^*^	(+4.8;+9.9)	+6.9^*^	(+1.9;+12.2)
Cervix uteri	-4.2^*^	(-6.6;-1.7)	-1.8	(-5.7;+2.3)	-5.9^*^	(-9.4;-2.3)	-0.6	(-4.5;+3.4)
Ovary	-2.1	(-6.7;+2.8)	-0.0	(-6.1;+6.4)	-7.5^*^	(-11.5;-3.3)	+0.8	(-5.1;+6.9)
Kidney	-3.4	(-11.1;+4.9)	+0.1	(-12.7;+14.7)	-0.9	(-6.7;+5.2)	+5.1	(-9.4;+22.1)
Bladder	-4.4	(-15.4;+8.0)	+0.1	(-21.8;+28.1)	-10.4	(-26.3;+9.0)	+4.0	(-5.2;+14.1)
Thyroid	+5.3^*^	(+2.8;+7.9)	+9.8^*^	(+5.6;+14.2)	+5.0^*^	(+1.8;+8.4)	-0.8	(-5.3;+3.8)
Hodgkin lymphoma	+4.7	(-1.3;+11.1)	+3.2	(-1.6;+8.3)	-1.2	(-9.3;+7.6)	+23.9	(-4.1;+59.9)
Non Hodgkin lymphomas	+2.9	(-1.0;+6.9)	+6.3^*^	(+0.9;+12.0)	+4.1	(-1.1;+9.6)	-2.1	(-8.1;+4.3)
Leukaemias	+3.1	(-3.9;+10.6)	+11.2^*^	(+2.9;+20.1)	-0.1	(-7.4;+7.8)	+3.6	(-5.3;+13.4)
Unspecified and ill-defined cancers	-0.4	(-5.9;+5.4)	-5.4	(-11.0;+0.6)	-3.2	(-9.1;+3.0)	+4.6	(-2.8;+12.7)
All cancers but skin	+3.7^*^	(+2.6;+4.8)	+5.2^*^	(+3.1;+7.4)	+2.8^*^	(+1.6;+4.0)	+4.4^*^	(+2.5;+6.4)

^*^p < 0.05.

In men, stomach cancer incidence rates decreased continuously during the whole period. Nasopharyngeal incidence rates increased up to 1996–2000 and declined thereafter. Non-Hodgkin’s lymphomas rates increased up to 1996–2000 and leveled-off thereafter. Cancers of gallbladder and kidney, Hodgkin lymphomas, leukemias and ill-defined cancer showed somewhat stable trends during the 25 years examined. Liver cancer rates increased since 1996–2000 after a decrease. Conversely, increased trends were observed for cancer of the colon-rectum, larynx, lung, prostate, bladder and thyroid (since 1996–2000).

In women, stomach cancer rates declined up to 1996–2000 and leveled-off thereafter, whereas rates of invasive cervical cancer increased up to 1991–1995 and declined thereafter. Gallbladder rates increased up to 2001–2005 and declined thereafter while lung cancer incidence rates increased after 2001–2005. Cancers of nasopharynx, larynx, ovary, kidney, and bladder, Hodgkin’s lymphomas, leukemias and ill-defined cancers showed somewhat stable trends during the 25 years examined. Liver cancer rates increased since 1996–2000 after a decrease. By contrast, cancer of the colon-rectum, breast, and thyroid, and non-Hodgkin’s lymphomas increased throughout the whole period examined.

Furthermore, between 1996 and 2010, cancer incidence rates showed some statistically significant negative APCs (% per year) (Table 2). Decreases were displayed for nasopharyngeal cancer (APC = -3.4) in men, and for gallbladder (-3.2), and cervical cancers (-4.2) in women. Many statistically significant positive APCs (Table 2) were observed. Increases in both sexes were observed for colon-rectum (APC = +5.4 in men and +4.5 in women), liver (APC = +8.9 in men and +5.4 in women), and lung (APC = +1.8 in men and +10.0 in women). Men displayed increases for bladder (APC = +5.9), and prostate (APC = +4.3) cancers, while women displayed increases for larynx (APC = +13.8), breast (APC = +8.2), and thyroid (APC = +5.3) cancers.

Some variability in temporal trends was registered according to sex, age groups and cancer site/type. However, this variability was partially compatible with random variation due to small number of cases in the considered strata. The 15–44 age group displayed statistically significant decreases for nasopharynx carcinoma (APC = -4.9), and stomach cancer (APC = -7.0) in men, and for stomach (APC = -6.6), and gallbladder (APC = -8.7) cancers in women. Increases were observed for leukemias (APC = +13.7) in men, for cancers of the breast (APC = +9.7), and thyroid (APC = +9.8) and non-Hodgkin lymphomas (APC = +6.3), and leukemias (APC = +11.2) in women. The increase of leukemias incidence was mainly related to the increase of lymphoid leukemias, however no complete information was available about the type of lymphoid leukemia (acute, chronic, or other). The 45–64 age group displayed statistically significant decreases for nasopharynx carcinoma (APC = -4.0), in men, and for cervical (APC = -5.9) and ovarian (APC = -7.5) cancers in women. Increases were observed for colon-rectum (APC = +3.7), and bladder (APC = +3.5) cancers in men, and for colon-rectum (APC = +5.3), lung (APC = +10.4), breast (APC = +7.3), and thyroid (APC = +5.0) cancers in women. The 65+ age group displayed exclusively statistically significant positive APCs for colon-rectum (APC = +11.1), lung (APC = +2.8), prostate (APC = +5.0), and bladder (APC = +8.0) cancers in men, and for colon-rectum (APC = +7.5), lung (APC = +7.8), and breast (APC = +6.9) cancers in women.

We investigated cancer incidence trends recorded by the Cancer Registry of the Wilaya of Setif, Algeria, across a 25 year period (1986–2010). To the best of our knowledge, this is the first report to investigate a large number of different types of cancer according to age for a quarter century in a northern African population. Decreasing trends were observed for nasopharyngeal carcinoma in men, and for cervical and gallbladder cancers in women, whereas increasing ones were observed mainly in leading cancers both in men (lung, colon-rectum, prostate, liver, and bladder) and women (breast, colon-rectum, lung, liver, thyroid and larynx).

Leading cancers were similar to those reported by other North-African studies [2–4, 6, 13, 14]. In contrast with the United States and other Western Countries, lung, colon-rectum, female breast, and prostate cancers showed in Setif increasing temporal trends [9]. The observed temporal trends give clues about large scale changes in exposure to risk factors, and to improvements in opportunistic early diagnosis.

Lung cancer incidence is largely determined by prevalence and duration of smoking. In Algeria, between 1970 and 1980, prevalence of smoking increased both for blond (34 times) and for black (+50%) tobacco. In addition between 1978 and 2005 prevalence of smoking increased from 7.7% to 28.6% [3, 15, 16]. Smoking is also strongly associated with the occurrence of bladder, kidney, and larynx carcinomas. The increasing trends in incidence of all these cancers in men can thus be ascribed to the increased prevalence of smoking over time. In women, the smoking effect is noticeable only for lung and larynx cancers and an increase of incidence of bladder and kidney cancers is likely expected. The sharp temporal variations observed in laryngeal cancer in women across age groups are compatible with random variations linked to small number of incident cases.

Nasopharyngeal carcinoma (NPC) is a rare malignancy in most regions of the world, but it is endemic in Algeria and more generally in North Africa. Major risk factors include Epstein-Barr virus infection, genetic susceptibility, consumption of salt-preserved fish or other preserved foods, a family history of NPC, and tobacco smoking [17]. In Setif, traditionally preserved food such as “harissa”, pickled fruits and vegetables, dried and salted meat or fat, rancid butter, and domestic fumes have been described as NPC risk factors [16, 18–20]. By contrast XRCC1 and hOGG1 genes are unlikely to play a role in the susceptibility to NPC in North Africans [21]. We observed that NPC decreased in men but not in women. However, which determinants reflected this different pattern across sexes remains outside the scope of an observational study.

Changes in viral hepatitis prevalence rather than alcohol consumption seemed to explain the rise of liver cancer incidence because the other alcohol-related cancers (oral cavity, pharynx or esophagus cancers) were stable, with ASR-WRs below 1/100.000 (not shown). The prevalence of hepatitis B virus in blood donors was around 3.6% in 1989 [3]. To the best of our knowledge, no other information such as prevalence of hepatitis C virus is yet available.

Breast cancer, the most common female cancer diagnosed in Setif, dropped in all age groups. The reasons for this shift are unknown, but they may include increases in the prevalence of risk factors that modify endogenous levels of sex hormones such as early menarche, late menopause, late childbearing, having fewer children, and low prevalence of breastfeeding, and unhealthy diets, physical inactivity or obesity [2, 22]. For instance, total fertility (average number of children per woman) declined in the last 30 years in Algeria (7.18 children per woman in the 1975–80 period to 2.72 in 2005–2010 period) [23]. Moreover, in two neighboring countries of Algeria, Morocco and Tunisia, obesity is significantly higher among women than among men (22.7% vs. 6.7% in Tunisia and 18% vs. 5.7% in Morocco) and prevalence of obesity among women has tripled over the past 20 years [24]. Half of all women are overweight or obese (BMI > 25) with 50.9% in Tunisia and 51.3% in Morocco [24]. BRCA1 and BRCA2 mutations have been identified in Algerian patients. The pathogenic mutations observed are mainly similar to those described in European counties except one found in a family of Kabylia, a northern neighboring region of Setif [25]. Noteworthy, endometrial and ovarian cancers, which share risk factors with breast cancer, did not display increases. In particular, ovarian cancer displayed a decrease in the 45–64 years age group. It is possible that opportunistic mammography anticipated the diagnoses of breast cancer in younger age groups.

Invasive cervical cancer (ICC) is the third most common female cancer in Setif, and a sharp decline in ICC incidence was observed mainly in the 15–44 age group. A potential shift of ICC diagnoses to uterus, unspecified (near 16% of all uterine cancers in 2006–2010) cannot explain this decline. The development of cervical cancer is causally associated to infection by high-risk Human Papilloma Viruses (HPV). In Algeria, the HPV16 in single- or multiple-type infection has been shown to be the predominant HPV type (61.4%) in ICC, followed by HPV18 (15.8%), and HPV45 (6.4%) [26]. The prevalence of HPV16 is similar to the prevalence observed in European or North-American populations [26]. In Setif, the prevalence of high-risk HPV was 75%. Moreover, in all age groups a drop of the squamous cell carcinoma (SCC) histologic subtype (89% of all ICC diagnoses in 1986–1990, vs. 53% in 2006–2010) is observed. This decrease can thus be ascribed to opportunistic early detection by cytological screening that is more effective in detecting SCC than adenocarcinomas [27]. A similar pattern was observed in the Center of Tunisia after the introduction of regional cytological screening program [4].

Thyroid cancer (TC) incidence increased in women in 15–44 and 45–64 age groups. In 2006–2010, the most common form of TC was the papillary sub-type and was related to most of the increased incidence (19% of all TC diagnoses in 1986–1990, vs. 49% in 2006–2010). This is thought to be largely due to increased detection of small papillary cancers associated to widespread use of ultrasound and fine needle biopsies [28].

Prostate cancer is the third most common cancer in men in Algeria. A number of risk factors detected in Western Algeria can explain the increased trend in prostate cancer incidence, such as high consumption of red meat, and dairy products, or low consumption of olive oil, cauliflower, and intake of fruits [29]. Presently prostate cancer incidence is closely linked to increased uptake of serum prostate-specific antigen (PSA) testing in western countries, but no data on PSA testing are yet available to verify this hypothesis in Algeria.

Reduction of stomach cancer incidence is generally attributed to diets rich in fruit and vegetables, improved preservation of foods, and reduced prevalence of Helicobacter pylori [22]. Some discrepant trends across age groups between gastric cancer and NPC that share a common risk factor (preserved foods) may suggest a decreasing exposure to Helicobacter pylori in the 15–44 age group.

Colon-rectum cancer increased in 45–64 and 65+ age groups and is the 2^nd^ most common cancer in both sexes. The adoption of western lifestyles and behaviors such as consumption of high-fat diets and physical inactivity could explain the increase of these cancers together with genetic predisposition [3]. It is possible that opportunistic early detection contributed to the increase of incidence in older age groups.

Gallbladder is the 5^th^ most common cancer in Setif women. Algerian women show the highest rates of gallbladder cancer as compared to the other Arab countries where this cancer is generally rare [2]. The temporal trends showed a favorable pattern in the 15–44 age group suggesting that differential risk factors are acting.

The selective increase of non-Hodgkin’s lymphomas in the 15–44 age group in women is intriguing. Moreover, further studies are required to elucidate the increase of leukemias, particularly lymphoid leukemias, in the 15–44 age group.

Incidence data have potential limitations in accuracy and completeness that should be made clear. Firstly, cancer and comorbidities are extremely common in elderly people and the percentage of histologically confirmed cancers is generally lower among them than in younger age groups. Although under-diagnosis and under-registration seem to be unlikely, the complete identification of true cases in older age groups may be difficult. Secondly, the accuracy of DCOs depends on coding practices. The low number of DCO should have limited the possibility of differential practices influencing death certification procedures. Thirdly, no mortality/incidence ratios were available (as in all North-African cancer registries). This information would have given further confidence in the observed results. Fourthly, due to the relatively small population covered (1.5 million, i.e. 5% of Algeria’s population), more accurate results from a bigger cohort are needed.

Among the strengths of the study, MV% reached good or acceptable levels in the studied cancer sites. Moreover MV% did not change significantly over time, thus, any estimated trend in incidence is unlikely to be the consequence of changes in the degree of ascertainment. Moreover, a small proportion of cancer cases were reported by DCOs. Finally, the availability of complete morphology data provided the opportunity to associate some trends to potential early detection initiatives.

## Conclusions

Some main conclusions can be drawn from the trends of cancer incidence observed in the last 25 years in Setif. Besides ageing of the population, temporal variations in caseload indicate cohort effects due to differential exposure to risk factors and improved detection methodology. In the light of international recommendations against cancer [30] three leading priorities can be advocated. Firstly, in the light of the observed variation in incidence that may be linked to participation in opportunistic screenings, it is reasonable to promote organized population-based screening programs aimed at reducing breast, colon-rectum, and cervical cancers. Secondly, primary prevention intervention at population level must be established to attempt a reduction in factors such as tobacco smoking and/or overweight. Thirdly, efforts should focus on the reduction of vaccine-preventable cancers. HPV vaccination for the prevention of cervical cancer should be introduced. The use of hepatitis B virus (HBV) vaccine for prevention of hepatocellular carcinoma should also be expanded. However, the application of these international recommendations against cancer in Setif must take into account epidemiological transition, lifestyle and environmental changes, poor health education, and difficult access to health care facilities. Thus, using evidence-based medicine, AROME project defined the “minimal requirements” guidelines (vs. standard of care guidelines) to form a basis for the development of practices and policies for diagnosis and treatment in limited-resource countries. The guidelines included cancers of prostate, bladder, lung, breast, head and neck, colon-rectum, and cervix [31].
